# Proximity proteomics reveals unique and shared pathological features between multiple system atrophy and Parkinson’s disease

**DOI:** 10.1186/s40478-025-01958-5

**Published:** 2025-03-23

**Authors:** Solji G. Choi, Tyler R. Tittle, Raj R. Barot, Dakota J. Betts, Johnie J. Gallagher, Jeffrey H. Kordower, Yaping Chu, Bryan A. Killinger

**Affiliations:** 1 Department of Neurological Sciences, Rush University Medical Center, Chicago, IL USA; 2 ASU-Banner Neurodegenerative Disease Research Center and School of Life Sciences, Arizona State University, Tempe, AZ USA; 3 University of Michigan, Ann Arbor, MI USA; 4 University of Illinois at Chicago, College of Medicine, Chicago, IL USA; 5 Graduate College, Rush University Medical Center, Chicago, IL USA

**Keywords:** Neurodegeneration, Proximity proteomics, Antioxidant, Spatial omics, SNARE complex

## Abstract

Synucleinopathies such as Parkinson's disease (PD), dementia with Lewy bodies (DLB), and multiple system atrophy (MSA) are neurodegenerative diseases with shared clinical and pathological features. Aggregates of alpha-synuclein (αsyn) phosphorylated at serine 129 (PSER129) are hallmarks of synucleinopathies, which, for PD/DLB, are found predominantly in neurons, whereas in MSA, aggregates are primarily found in oligodendroglia. It remains unclear whether the distinct pathological presentations of PD/DLB and MSA are manifestations of unique or shared pathological processes. Using the in-situ proximity labeling technique of biotinylation by antibody recognition (BAR), we compared aggregated αsyn-interactomes (BAR-PSER129) and total αsyn-interactomes (BAR-MJFR1) between MSA (n = 5) and PD/DLB (n = 10) in forebrain and midbrain structures. Comparison between MSA and PD/DLB-enriched proteins revealed 79 PD/DLB-differentially abundant proteins and only three MSA-differentially abundant proteins (CBR1, CRYAB, and GFAP). Pathway enrichment analysis revealed that vesicle/SNARE-associated pathways dominated PD/DLB interactions, whereas MSA was strongly enriched for metabolic/catabolic, iron, and cellular oxidant detoxification pathways. A subnetwork of cytosolic antioxidant enzymes called peroxiredoxins drove cellular detoxification pathway enrichment in MSA. A network of 26 proteins, including neuronal-specific proteins (e.g., SYNGR3) with HSPA8 at the core, was shared between MSA and DLB/PD. Extracellular exosome pathways were universally enriched regardless of the disease or BAR target protein. In conclusion, synucleinopathies have divergent and convergent αsyn-aggregate interactions, indicating unique and shared pathogenic mechanisms. MSA uniquely involves oxidant detoxification processes in glial cells, while vesicular processes in neurons dominate PD/DLB. Shared interactions, specifically SYNGR3, between MSA and PD/DLB suggest that neuronal axons are the origin of both diseases. In conclusion, we provide αsyn protein interaction maps for two distinct synucleinopathies.

**Supplementary Information:**

The online version contains supplementary material available at 10.1186/s40478-025-01958-5.

## Introduction

Primary synucleinopathies are neurodegenerative diseases, including Parkinson's disease (PD), dementia with Lewy bodies (DLB), and multiple system atrophy (MSA), which have common pathological hallmarks, specifically alpha-synuclein (αsyn) aggregates [18]. The distribution and abundance of αsyn aggregates are highly heterogeneous between synucleinopathies, with copathologies (e.g., tau and beta-amyloid) also being common [9, 29]. MSA is unique among synucleinopathies because αsyn aggregates are predominantly observed in oligodendroglia (glial cytoplasmic inclusions or “GCIs”, also called Pap–Lantos bodies) [42]; in contrast, PD/DLB brain neurons exhibit the majority of Lewy pathology (neuronal inclusions or “NIs”) [18]. NIs are assumed to originate in neurons, but the origins of GCIs are less obvious because, unlike neurons, oligodendroglia do not normally, abundantly, express αsyn [2, 13, 37]. Therefore, in the MSA brain, either αsyn aggregates spread from diseased neurons to oligodendroglia or diseased oligodendroglia ectopically express αsyn. There is empirical evidence to support both hypotheses [48], but the details remain unclear.

Research has focused on characterizing NIs in PD/DLB brains, but there is less understanding of the GCIs in the MSA brain, partly due to the lower incidence of MSA (1.6 cases per 100,000 individuals over the age of 40) than PD/DLB [4, 44, 55, 62]. Recently, we applied an in situ proximity labeling technique called biotinylation by antibody recognition (BAR) to measure the αsyn interactome in the rodent brain and in the human PD/DLB brain and reported that vesicles, neurons, and neuronal synapses are strongly associated with αsyn and αsyn aggregates [27], which is consistent with the neuronal origin of PD/DLB. BAR has not been applied to other synucleinopathies; in this study, we utilized BAR to compare the αsyn interactomes of both diseases to define better core molecular features, distinguishing MSA and PD/DLB.

## Materials and methods

### Tissue preparation

Human brain tissues from individuals with a primary clinical diagnosis of MSA, PD, DLB, or Parkinson’s disease dementia (PDD) were obtained from the Rush Movement Disorders Brain Bank (see Table 1 for detailed case information). All brain samples were prepared following a previously described method [27]. Briefly, 2-cm coronal slabs were fixed by immersion in a 4% paraformaldehyde solution in 0.1 M phosphate-buffered saline (PBS) at pH 7.4 for seven days at 4 °C. The fixed slabs subsequently underwent gradual equilibration in a cryoprotectant mixture composed of PBS, 2% dimethyl sulfoxide, and 20% glycerol. The brain regions of interest were cut into 40 μm coronal sections via a freezing stage sliding knife microtome (American Optical). The brain sections were stored in a cryoprotectant solution at − 20 °C until further processing.

**Table 1:** Characteristics of MSA and PD/DLB patients

Sample #	Sex	Age at death (years)	Method of fixation	Hoehn and Yahr Scale	*Disease Duration (years)	Clinical diagnosis	Pathological diagnosis
1	M	64	Immersion fixation 7–10 days	NA	NA	MSA	MSA-C
2	M	55	Immersion fixation 7–10 days	NA	NA	MSA	MSA-P
3	F	75	Immersion fixation 7–10 days	4	4	MSA-P	MSA-P
4	F	65	Immersion fixation 7–10 days	5	10	MSA-P	NA
5	M	60	Immersion fixation 7–10 days	3	2	MSA-P	NA
6	M	74	Immersion fixation 7–10 days	NA	9	PD	Corticobasal degeneration with parkinsonism. Alzheimer’s
7	F	87	Immersion fixation 7–10 days	3	2	PD	LBD. Primary age related tauopathy
8	M	81	Immersion fixation 7–10 days	4	3	PD	PD-brainstem prominent
9	M	84	Immersion fixation 7–10 days	4	4	PD	NA
10	M	80	Immersion fixation 7–10 days	4	6	DLB	DLB diffuse subtype
11	M	79	Immersion fixation 7–10 days	4	8	PD	NA
12	F	89	Immersion fixation 7–10 days	2	4	PD	NA
13	M	80	Immersion fixation 7–10 days	4	8	PD	NA
14	M	73	Immersion fixation 7–10 days	2	11	LBD	NA
15	M	70	Immersion fixation 7–10 days	4	5	PD/PSP	NA

^*^The duration of the disease is defined as the period from the onset of symptoms to death, as documented in the clinical records of the Rush Movement Disorder program. “NA” denotes information not available

## Immunohistochemistry (IHC)

Free-floating brain sections were washed in dilution media (“DM,” 5 mM Tris–HCl pH 7.6, 150 mM NaCl, 0.05% Triton X100), followed by heat-induced antigen retrieval (HIAR) using sodium citrate buffer (10 mM sodium citrate, 0.05% Tween-20, pH 6.0) at 80 °C for 30 min. The sections were subsequently incubated in peroxidase quenching solution (0.3% hydrogen peroxide, 0.1% sodium azide) containing blocking buffer (3% goat serum, 2% bovine serum albumin (BSA), 0.4% Triton X-100 in DM) for 1 h at room temperature. The tissues were then incubated overnight with PSER129 antibody (Abcam, "EP1536Y", ab51253, RRID: AB_869973) or MJFR1 antibody (Abcam, ab138501, RRID: AB_2537217) diluted 1:50,000 or 1:20,000 in blocking buffer, respectively. The following day, the sections were washed in DM and incubated with a biotinylated anti-rabbit antibody (Vector Laboratories, BA-1000, RRID: AB_2313606) at a 1:200 dilution in blocking buffer for 1 h at room temperature, followed by rinsing in DM. The tissues were incubated with an elite avidin–biotin complex (ABC) reagent (Vector Laboratories, PK6100, RRID: AB_2336819) for 75 min at room temperature. The tissues were subsequently washed in DM and sodium acetate buffer (0.2 M imidazole, 1.0 M sodium acetate buffer, pH 7.2). Sections were developed via a standard nickel-enhanced 3,3'-diaminobenzidine (DAB)-imidazole protocol, rinsed again with sodium acetate buffer and PBS (50 mM Tris–HCl, pH 7.2, 158 mM NaCl), and then mounted on gelatin-coated glass slides. Counterstaining of the tissues was performed using methyl green. The sections were dehydrated, cleared with xylenes, and coverslipped with Cytoseal 60 (Fisher Scientific). The detailed IHC protocol can be found at protocols.io (https://doi.org/10.17504/protocols.io.8epv5x3mdg1b/v1).

## Microscopy and imaging

Prepared slides were imaged via an upright microscope (Olympus, BX53) to obtain images using a 4X objective. For 20X objective whole-section scans, either a Nikon A1 inverted microscope or Odyssey M imager (LI-COR) was utilized. The images underwent downsizing, cutting, autocolor balancing, and autobrightness adjustments via Adobe Photoshop (RRID: SCR_014199) to improve the figure and data presentation clarity. The edited images were imported into Adobe Illustrator (RRID: SCR_010279) for arrangement and figure construction.

## Biotinylation by antibody recognition (BAR)

For each case, PSER129 (BAR-PSER129), total αsyn (BAR-MJFR1), and a primary antibody omission negative control (BAR-NEG) were performed essentially as previously described [27]. A notable exception was that the tyramide reaction was conducted in 100 mL of borate buffer (0.05 M sodium borate, pH 8.5) containing 14.7 μL of stock biotinylated tyramide (Sigma, 12.5 mg/mL dissolved in DMSO) and 10 μL of hydrogen peroxide (30% H_2_O_2_, Sigma‒Aldrich) for 30 min at room temperature. After BAR labeling, the sections were washed with PBS and placed in crosslink reversal buffer consisting of 5% SDS, 500 mM Tris–HCl (pH 8.0), 2 mM EDTA, 1 mM PMSF, and 200 mM NaCl. The tissues were heated to 95 °C for 30 min and incubated at 65 °C overnight. The samples were centrifuged to remove insoluble debris, and the supernatant was diluted in TBST (150 mM NaCl, 1% Triton X-100, and 50 mM Tris–HCl, pH 7.6). Biotinylated proteins were captured using 40 μL of streptavidin-coated magnetic beads (Thermo Fisher, Catalog No. PI88817) at room temperature for two hours. The beads were isolated using a magnetic stand (Millipore), washed with excess TBST three times for 30 min each, and incubated overnight for 16 h at 4 °C. Following the pulldown, the lysates were retained for later characterization via western blotting. The capture proteins were eluted by heating the beads to 98 °C in 50 µL of sample buffer containing 1X LDS sample buffer (Invitrogen, Cat. B0008) and 1X sample reducing agent (Invitrogen, Cat. B0009) for 10 min. A total of 40 µL of each sample was run on a 4–12% Bis–Tris Gel (Fisher Scientific, Cat. NW04127BOX) for ~ 7 min at 200 V (run until the sample entered the gel). The gel was incubated overnight in fixation buffer (50% ethanol and 10% acetic acid). The next day, the gel was rehydrated with ultrapure water and stained with Coomassie blue (Invitrogen, LC6060). The entire lane containing the captured protein was excised for Liquid chromatography-tandem mass spectrometry (LC–MS/MS) analysis.

## Liquid chromatography-tandem mass spectrometry (LC–MS/MS)

The samples were prepared and analyzed via LC‒MS/MS via optimized protocols as previously described [28]. The gel pieces containing the embedded proteins were digested with trypsin according to optimized protocols. The resulting tryptic peptide mixture was then subjected to mass spectrometry.

## Determination of BAR-enriched proteins

Combining multiple search engines and quantitative software can improve the analysis of MS-based proteomics data sets [56]. Therefore, we combined two common approaches, namely, Mascot/Scaffold (i.e., total normalized spectra, “TNS”) and Andromeda/Maxquant (LFQ). Raw files were independently processed and analyzed using each approach (see Additional files 12 and 13 for parameter details). Differential abundance (DA) analysis was performed using the R package DEP designed for proteomics analysis [71]. Proteins were kept if they were identified in all replicates of at least one of the experimental group, the data were normalized via variance stabilizing transformation, and the QRILC method imputed missing values. Significant proteins were identified using protein-wise linear models and empirical Bayes statistics using Limma. Proteins from each analysis were combined for downstream pathway analysis. False discovery rate (FDR) p values were adjusted to account for multiple tests, and proteins with q values less than 0.05 were deemed significant. These values were estimated via Fdrtool. Proteins significantly enriched for each capture (i.e., significantly enriched over BAR-NEG) were exported and analyzed by multiple list comparator (Molbiotools.com) to determine overlapping and unique proteins identified for each condition. Overlapping proteins were determined for all conditions (i.e., BAR capture and disease state, Fig. 3C), and individual comparisons were made within each capture condition (Figs. 4 and 5). Direct comparisons were also made between PD/DLB and MSA for each BAR capture, with any known background proteins (i.e., significantly enriched in BAR-NEG) deemed insignificant.

## Enrichment mapping

Protein lists were input into the gProfiler web user interface. Enrichment analysis was conducted without ranks or weighting because we cannot assume that measured protein abundance is a function of distance to BAR target or relevance. The Gene Ontology (GO) and Reactome databases were queried without electronic annotations. To reduce redundancy and minimize the size of the enrichment map, gProfiler Driver terms were exclusively used to construct the final enrichment map. The enrichment results were exported to Cytoscape and plotted via Enrichmentmap V 3.4.0 with Autoannotate.

### STRING

Protein lists were imported into the STRING version 12.0 web user interface. The search was conducted with *H. sapiens* as the reference organism. Mapped proteins were then used to generate STRING functional or physical protein–protein interaction networks. The minimum required interaction score was set to 0.5 unless otherwise noted. STRING maps were then exported to Cytoscape version 3.10.2 for MCL clustering, pathway enrichment, network analysis (CentiScaPe 2.2), and network layout configuration.

## Spot blotting

Before conducting LC–MS/MS analysis, 1 µl of streptavidin bead eluent was applied to a methanol-activated polyvinylidene difluoride (PVDF) membrane and fully dried. The dried membrane was then reactivated with methanol, followed by washing with ultrapure water and post-fixation in 4% paraformaldehyde for 30 min. The blots were subsequently rinsed with TBST (20 mM Tris–HCl pH 7.6, 150 mM NaCl, 0.1% Tween-20) and immersed in blocking buffer (TBST supplemented with 5% BSA or 5% dry milk) for 1 h at room temperature. To detect biotinylated proteins, the blots were incubated with prepared ABC reagent (Vector Laboratories) in blocking buffer for 30 min at room temperature. For αsyn detection, the blots were incubated overnight with the anti-αsyn antibody SYN1 (BD Lifesciences, #610,787, RRID: AB_398108) diluted 1:2000 in blocking buffer. After incubation, the blots were washed and incubated with anti-mouse HRP conjugate (Cell Signaling Technology, CST7076S, RRID: AB_10956588) diluted 1:6000 in blocking buffer. Following another round of washing with TBST, all membranes were imaged using enhanced chemiluminescence (ECL) substrates (Bio-Rad, product # 170–5060) and a ChemiDoc imager (Bio-Rad).

## Western blotting

10 µg of protein from the bead eluent was loaded on 4 to 12% gradient Bis‒Tris gels (Thermo Fisher) and run until the loading dye reached the bottom of the gel. Resolved proteins were blotted onto the PVDF membrane using wet transfer at 100 V for 1 h. Following transfer, the membranes were rinsed with ultrapure water (18Ω) and then fixed with 4% paraformaldehyde for 30 min at room temperature. Membranes were dried completely and reactivated with methanol. The gels were then stained with Revert total protein stain (LI-COR, product # 926–11,016) according to the manufacturer's protocol and imaged using an Odyssey M imager (LI-COR). Membranes were then rinsed in TBST and blocked in TBST containing 5% BSA or dry milk for 1 h at room temperature. Primary antibody incubation was performed with SYN1 (BD Lifesciences, dil. 1:2000) or PSER129 (Abcam, dil. 1:50,000) diluted in blocking buffer overnight at 4 °C. The next day, the membranes were washed in TBST and incubated for 1 h with either anti-rabbit (dil. 1:20,000) or anti-mouse (dil. 1:6,000) HRP-conjugated secondary antibodies (Cell Signaling Technology) diluted in blocking buffer. The membranes were washed and developed using a chemiluminescence (ECL) substrate (Bio-Rad) with a ChemiDoc imager (Bio-Rad). For quantitative blots, chemiluminescent blots were developed via the “optimal autoexposure” method of Bio-Rad, which involves generating a quantitative image.

## Quantitative analysis of the blots

The mean abundance of PSER129 or αsyn for western and spot blots was quantified via ImageJ (version 1.54 h, https://imagej.net/ij/download.html, RRID:SCR_003070). The data were then normalized to the total protein amount, or the fold enrichment was calculated based on negative controls. Statistical analysis and graphing were performed using GraphPad Prism (version 10.2.0, https://www.graphpad.com/, RRID: SCR_002798). One-way ANOVA with Tukey's post hoc test unless otherwise noted or two-tailed unpaired t-test was used to compare experimental groups.

## Quantification of IHC

Brightfield images were captured using an inverted confocal microscope equipped with a 20X objective (Nikon A1R). Annotation of each tissue section was performed within a bounding box of 2000 × 2000 pixels. All images underwent auto-exposure and auto-white balance adjustments using NIS-Elements software. RGB-based color thresholding was initially fine-tuned to quantify dark black pixels (i.e., PSER129 signal). This adjusted threshold was recorded using the Macro function in NIS-Elements and then uniformly to all images, irrespective of the disease (MSA or PD/DLB) or the region of the brain (WM or GM in the midbrain or forebrain) (Additional file 1: Figure S1 for the raw images and thresholding examples). The percentage area of the thresholded signals was subsequently exported and graphed using GraphPad Prism. The statistical analysis was performed using either One-Way or Two-way ANOVA with Tukey's multiple comparisons test unless otherwise noted. Matching between conditions was used for multiple measurements within the same case.

## Results

**Fig. 1:**
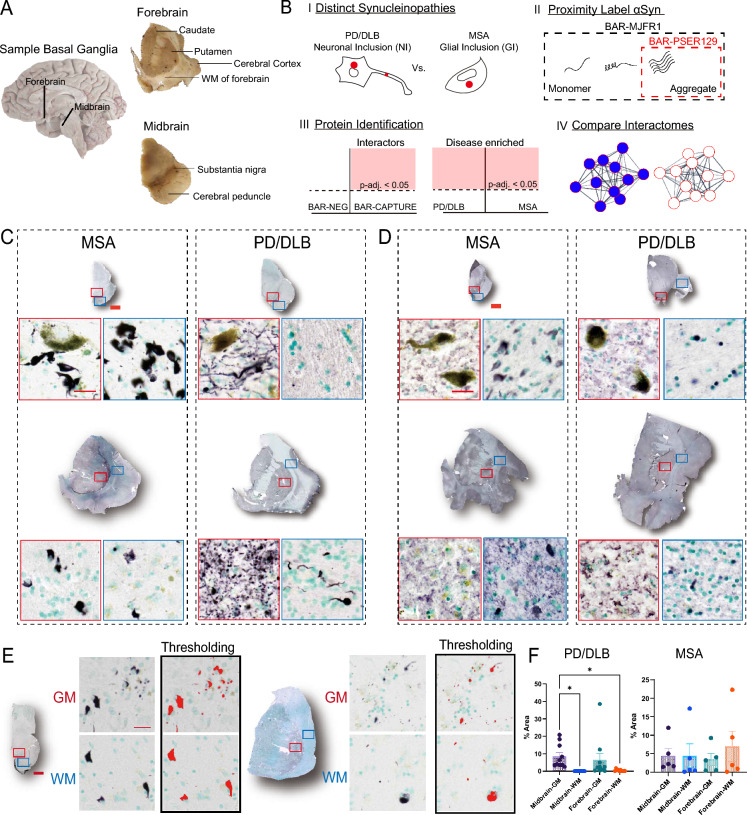
Summary of approach. **A** Depiction of specimen sampling for studies. Longitudinal image of the right hemisphere of the human brain with the approximate location of sampling sites shown (black lines). For BAR, a single coronal section through the Caudate Nucleus and Putamen (Forebrain) and a single transverse section through the midbrain were pooled and used. **B** Depiction of studies. The distribution of αsyn aggregates is distinct between the synucleinopathies PD/DLB and MSA. For PD/DLB, αsyn aggregates are observed prominently in neuronal cell bodies and projections, termed neuronal inclusions (NI). In contrast, in the MSA brain, αsyn aggregates occur prominently in glia, termed glial inclusions (GI). Biotinylation by antibody recognition (BAR) was used to identify and compare interactomes of total αsyn (BAR-MJFR1) and aggregated αsyn (BAR-PSER129) directly in the PD/DLB and MSA brain. MJFR1 antibody maps to an epitope of a.a. 118–123 of αsyn’s c-terminus and captures physiological monomeric forms and aggregates. PSER129 preferentially labels αsyn aggregates, especially in the postmortem brain where physiological PSER129 is scarce [8], and thus, BAR-PSER129 will capture aggregate interactions. LC–MS/MS identified BAR-labeled proteins and differential abundance analysis was used to determine interactors (i.e., proteins enriched over BAR-NEG) and disease-enriched proteins (PD/DLB vs. MSA). The resulting proteins were analyzed using a combination of approaches: overlap analysis, pathway enrichment mapping, and protein interaction network mapping. IHC staining for **C** PSER129 and **D** αsyn (i.e., MJFR1) in forebrain and midbrain sections. Sections were stained using nickel-DAB chromogen (black) and counterstained with methyl green (green). Whole-section scans and high-magnification images of select pathology-bearing regions are shown with red and blue boxes denoting the approximate area of the high-magnification image. Signal thresholding was applied to 20X images of PSER129-stained tissues, specifically in gray matter (GM, red box) and white matter (WM, blue box) in the forebrain and midbrain for quantification. Enlarged 20X images showing the application of thresholding **E** and the subsequent quantification of PSER129 immunoreactivity across all cases **F**. Scale bars: C, D = 2 mm and 25 µm; E = 2 mm and 20 µm. MSA, n = 5; PD/DLB, n = 10.(mean ±SEM  *Tukey’s multiple comparisons test *p-adj. < *0.05)

To perform all studies, we used 5 clinically diagnosed MSA and 10 clinically diagnosed PD/DLB cases (See Table 1 for case details). Brain hemisections of the nigrostriatal pathway were used for all studies (Fig. 1A) because both brain regions are involved with the core clinical motor features of both MSA and PD/DLB, and the basal ganglia bears αsyn aggregates for these synucleinopathies. Tissue sections were positionally matched for all cases and consisted of forebrain regions (e.g., putamen, caudate, adjacent cortical regions) and the midbrain (e.g., substantia nigra).To compare MSA with PD/DLB, we performed in situ proximity labeling techniques BAR-MJFR1 and BAR-PSER129 [27], which capture total αsyn and aggregated αsyn spatially (~ 50–100 nm radius [52])-associated proteins, respectively (Fig. 1B). BAR-identified proteins were used to describe interactomes and compare the molecular characteristics of these two distinct synucleinopathies.

## αSyn in the MSA and PD/DLB forebrain and midbrain

Tissues were first characterized by IHC. Striatal containing forebrain and midbrain hemisections were stained for PSER129 and total αsyn (MJFR1). Results show widespread PSER129 pathology throughout the forebrain and midbrain sections (Fig. 1C). For PD/DLB cases, PSER129 pathology was dense in gray matter with substantia nigra, caudate, putamen, and the insular lobe was often also severely affected. In contrast, MSA cases had widespread PSER129 pathology in the white matter tracts of the midbrain and forebrain, consistent with known white matter involvement for MSA [12, 64] (Fig. 1C). The morphology of PSER129-positive structures in the PD/DLB and MSA brains was consistent with prominent NIs or GCIs [18, 42], respectively. MSA and PD/DLB exhibited αsyn (MJFR1) reactivity throughout the forebrain and midbrain sections, predominantly in the gray matter (Fig. 1D). GCIs were evident in the white matter because αsyn is of low abundance in white matter tracks of the healthy mammalian brain [17], making αsyn bearing oligodendroglia visible, even at low magnification and with total αsyn staining. In contrast, the NIs of PD/DLB observed with PSER129 staining were obscured by a strong ubiquitous total αsyn signal throughout the gray matter (Fig. 1D). Despite the apparent regional specificity of PSER129 staining for MSA and PD/DLB within some cases, a quantitative comparison of PSER129 between white and gray matter revealed high heterogeneity between cases and few overall significant differences in PSER129 abundance between MSA and PD/DLB (Fig. 1E, F). For PSER129 abundance in PD/DLB midbrain, significant differences (Tukey’s post-hoc test *P < *0.05) were observed between gray and white matter (Fig. 1F). Collectively, these results highlight the presentation of αsyn pathology and distribution of BAR targets for the cases used for these studies.

## BAR capture in MSA and PD/DLB forebrain and midbrain tissues

**Fig. 2:**
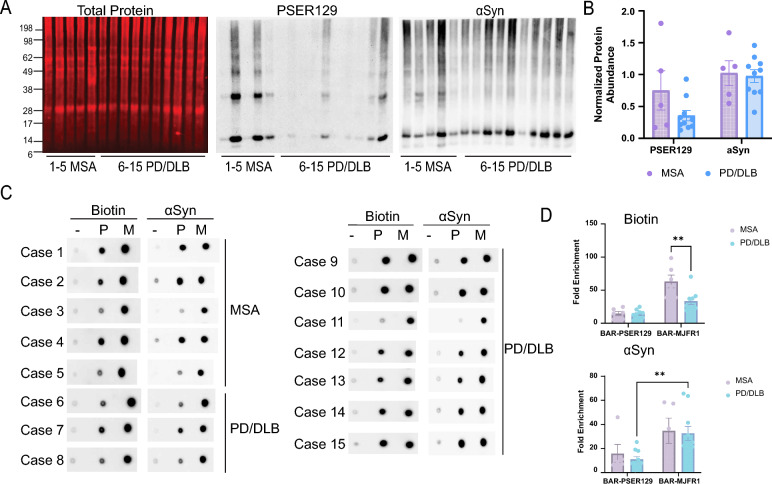
BAR capture in the PD/DLB and MSA brain. **A** 10 μg tissue lysate protein for each case were separated on 4–12% Bis–Tris gel transferred onto PVDF and stained for total protein (Revert Total Protein Stain, LI-Cor), PSER129, or αsyn. Chemiluminescence was used to detect PSER129 and αsyn. **B** PSER129 and αsyn relative density values were first normalized to total protein (i.e., loading control) and then normalized to the mean intensity for each group. **C** Spot blots of 1 µl eluent from BAR captures, including BAR-NEG (Primary antibody-omission control, “-”), BAR-PSER129 (aggregates “P”), and BAR-MJFR1 (total αsyn “M”) for each synucleinopathy case. Blots were probed for either biotin (ABC reagent) or αsyn (BDSYN1). **D** BAR enrichment was calculated by dividing for “P” and “M” by the relative density value for “-” (i.e., fold-enrichment over background) (mean ± SEM, Sidak's multiple comparison,**p-adj. < 0.005, n = 5–10)

After verifying the distribution and morphology of the αsyn pathology consistent with PD/DLB and MSA, BAR was performed as previously described [27] to capture proteins within proximity (predominantly within ~ 50–100 nm radius [53]) to PSER129 (i.e., αsyn aggregates) and αsyn. For each case, a positionally matched forebrain and midbrain section were pooled (Additional file 1: Figure S2 average of 81.7 ± 0.021 mg combined wet mass). Western blot (WB) analysis of proteins extracted from pooled forebrain and midbrain hemisections revealed that PSER129 and αsyn abundance was variable between cases (Fig. 2A, B), which is in agreement with the results of IHC (Fig. 1F); however, PSER129 or αsyn abundance was not significantly different between PD/DLB and MSA (Fig. 2B, PSER129; two-tailed unpaired t test, t(13) = 1.656, *p* = 0.1215; αsyn two-tailed unpaired t test, t(13) = 0.2505, *p* = 0.8061). High-molecular-weight species (> 17 kDa) were observed on WB for PSER129 and αsyn, which may be SDS-resistant aggregates [6] or residual formalin crosslinks [25]. αSyn truncation/cleavage products were also observed for most samples, which have been widely reported in healthy and diseased tissues [6, 26].

Following BAR, dot blots of BAR-captured proteins (i.e., bead eluent) revealed biotin and αsyn enrichment were similar between BAR captures for MSA and PD/DLB brains (Fig. 2C, D). Biotin enrichment was significantly greater for BAR-MJFR1 than for BAR-PSER129, regardless of disease state (Fig. 2D, two-way ANOVA, F (1, 13) = 47.68, *P < *0.0001). Biotin content was greater for BAR-MJFR1 capture in the MSA brain than in the PD/DLB brain (Fig. 2D, Sidak’s multiple comparison, p-adj.  = 0.0013). For PD/DLB, αsyn enrichment was significantly more in BAR-MJFR1 than BAR-PSER129 (Fig. 2D, two-way ANOVA F(1,13) = 13.64, P = 0.0027, Sidak’s multiple comparison, p-adj. * = *0.0092). Together, these results demonstrate successful BAR-PSER129 and BAR-MJFR1 enrichment for all cases in this cohort.

## BAR-identified αsyn and PSER129 interactomes in the MSA and PD/DLB brain

**Fig. 3:**
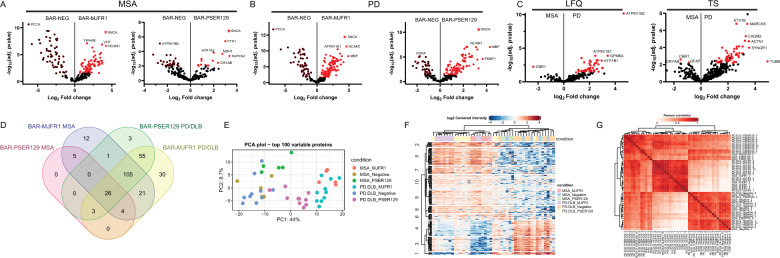
PSER129 and αsyn proximal proteins in the synucleinopathy brain. BAR-labeled proteins were identified by LC–MS/MS and quantified using two approaches Maxquant/andromeda label free quantification (LFQ) and Scaffold/mascot total normalized spectra values (TNS). Volcano plots comparing protein abundance (i.e., LFQ value) between capture (BAR-MJFR1 and BAR-PSER129) and assay background (BAR-NEG) for **A** MSA and **B** PD/DLB. αsyn (i.e., BAR target protein) is denoted as a star. All significant differentially abundant proteins appear red. LFQ results shown, TNS results can be found in Additional file. Names of select high abundance proteins are annotated. **C** BAR-PSER129 differentially abundant proteins between MSA and PD/DLB brain. Proteins previously found to be significantly enriched in background **A**, **B** were excluded for MSA with PD/DLB comparison. Both LFQ and TNS results are shown. **D** Venn diagram showing BAR enriched proteins (i.e., significant over BAR-NEG) for each BAR condition and disease state. Proteins from both analysis (LFQ and TNS) were included. **E** Principal component analysis (PCA) plot of the top 100 variable proteins (Additional file 1: See Figure S9 for TNS PCA plot). **F** Heatmap with non-biased hierarchical clustering of protein abundance across all samples. LFQ shown, for TNS see Additional file 1: Figure S7 **G** Correlation heatmap comparing protein abundances between all samples. LFQ shown, for TNS see Additional file 1: Figure S8. PD/DLB, n = 10. MSA, n = 5. MJFR1 from BAR-NEG in both MSA and PD/DLB brains, with close grouping observed within each disease state (Fig. 3E). Similar to BAR-NEG, BAR-PSER129 in MSA brain was more related to PC2 than PC1

After filtering missing proteins, 440 (TNS method) and 209 (LFQ method) proteins were quantified. In the MSA brain, 38 BAR-PSER129-enriched proteins and 175 BAR-MJFR1-enriched proteins were significant over background (i.e., BAR-NEG). In the PD/DLB brain, we identified 194 BAR-PSER129-enriched proteins and 245 BAR-MJFR1-enriched proteins. The BAR target αsyn (SNCA) was the most enriched protein for all captures when quantified by the LFQ method (Fig. 3A and B). For the TNS method, SNCA was enriched over the background but was not the most abundantly enriched capture protein (Additional file 1: Figure S3). BAR-NEG enrichment was observed for all the contrasts, and the known background biotin-binding protein propionyl-CoA carboxylase subunit alpha (PCCA) [1] was often most abundant in BAR-NEG (Fig. 3A, B). For BAR-MJRF1, a direct comparison between MSA and PD/DLB did not reveal any differentially abundant proteins. In contrast, for BAR-PSER129, direct comparisons revealed numerous proteins more abundant in PD/DLB, including ATP6V1B2, STX1B, and MARCKS (Fig. 3C). Three proteins (CBR1, GFAP, CRYAB) were differentially abundant in MSA (Fig. 3C). Overlap of identified proteins was greatest (105 proteins, 39.6% of all proteins) between groups excluding BAR-PSER129 in the MSA brain (Fig. 3D). Remarkably, despite the prominent GCI abundance observed in our MSA samples, not a single unique protein was identified for BAR-PSER129 in the MSA brain (Fig. 3D). However, BAR-MJFR1 and BAR-PSER129 identified proteins unique to MSA, including HSPA1B, PRDX1, SERTIN8, GSTP1, and PRDX2. Unique proteins were also identified for BAR-MJFR1 MSA, including PHGDH, TPM3, PFN1, HSPA5, ACOT7, CD47, IGHG3, UBE2N, GMFB, AQP1, NDUFA12, and EEF1A1. Identifying unique proteins was minimal in the MSA brain, and most proteins overlapped with PD/DLB (BAR-MJFR1 and BAR-PSER129). The principal component analysis (PCA) plot of the top 100 BAR-captured proteins showed the separation of BAR-MJFR1 from BAR-NEG in both MSA and PD/DLB brains, with close grouping observed within each disease state (Fig. 3E). Like BAR-NEG, BAR-PSER129 in MSA brain was more related to PC2 than PC1.

Figure 3F and G heatmaps show the hierarchical clustering of protein abundance (LFQ) and hierarchical clustering of Pearson correlations between all tested samples, respectively. Cases formed two main clusters (Fig. 3F), with one cluster having a strong “on-target” signal, containing the majority of BAR-MJFR1 captures and BAR-PSER129 captures in the PD/DLB brain. The second main cluster comprised background samples (BAR-NEG for MSA and PD/DLB) and BAR-PSER129 captures in MSA and PD/DLB brains showing weaker “on-target” signals. Regarding correlations, BAR-MJFR1 and BAR-NEG were closely correlated between MSA and PD/DLB, whereas BAR-PSER129 was less correlated between MSA and PD/DLB. (See Additional files 2-4 for results and 9,10 for input files).

## Pathway enrichment maps for MSA and PD/DLB

**Fig. 4:**
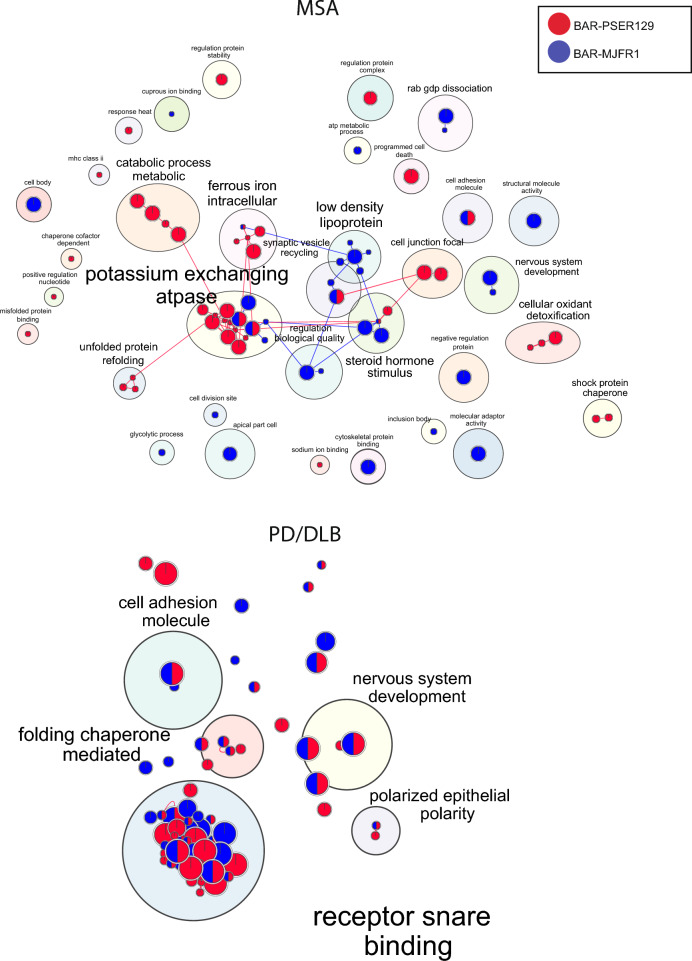
Pathway enrichment map for BAR-identified proteins. BAR-identified proteins from PD/DLB and MSA brains were analyzed by gProfiler, and significant (*q > *0.05) GO Driver Terms were mapped using Enrichmentmap and annotated with Autoannotate. GO Driver terms were used to reduce redundancy and simplify enrichment maps. Nodes are color-coded according to BAR capture condition, BAR-PSER129 (Red) or BAR-MJFR1 (Blue). Node size is proportional to the number of protein set size and edges denote pathway overlap size. The top-panel is MSA enrichment map, and bottom-panel is PD/DLB enrichment map

Next, we performed pathway enrichment analysis and identified hundreds of significantly enriched pathways (p-adj. < 0.05) for each experimental group (see Additional files 5-8 for enrichment files). To reduce the redundancy of identified pathways, we used the gProfiler “go driver term” function, which first groups significant terms by GO relation and then uses a simple greedy search strategy to identify leading gene/protein sets [30, 47]. The resulting top 10 driver terms are shown in Additional file 1 Figure S4, and all driver terms were used to construct the enrichment map in Fig. 4. For both MSA and PD/DLB, many clusters contained pathways enriched for both BAR captures, however unique pathway enrichment was observed for BAR-PSER129 and BAR-MJFR1. For MSA, BAR-PSER129's unique major clusters included “catabolic/metabolic processes,” “unfolded protein refolding,” “cellular oxidant detoxification,” “cell junction focal,” and “shock protein chaperone” clusters. For PD/DLB, all major clusters identified contained BAR-MJFR1-enriched pathways; therefore, no BAR-PSER129-specific clusters were detected. “Receptor/SNARE binding” was the major cluster for PD/DLB.

## Protein interaction networks identified in the synucleinopathy brain

**Fig. 5:**
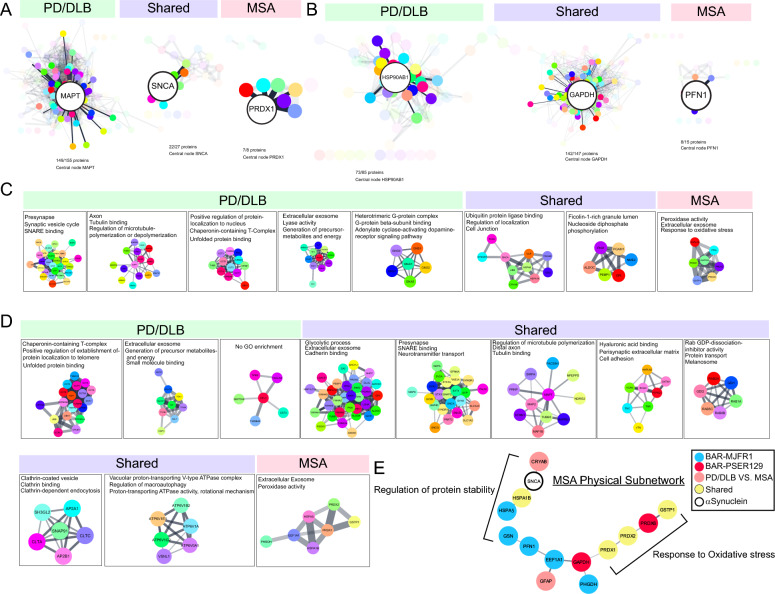
Protein interaction networks identified in PD and MSA brain. BAR identified proteins were analyzed with STRING to plot known functional interactions for each proximal proteome. Proteins were grouped according to whether they were unique to PD/DLB, MSA, or shared between synucleinopathies. STRING networks for **A** BAR-PSER129 and **B** BAR-MJFR1 are shown. CentiScaPe 2.2 was used to determine the central “driver” node for each STRING network (i.e., highest “betweenness” score). The driver node was enlarged, and the first neighbors in network highlighted. For, BAR-PSER129 **C** and BAR-MJFR1 **D** MCL clustering was used to group nodes. Clusters with 6 nodes or more are depicted. Top enrichment (GOCC, GOMF, GOBP) are annotated onto each cluster. **E** Physical interaction network of proteins specific to MSA for all differential expression analysis. BAR target SNCA was included in the network. Nodes annotated with top enriched pathway and disconnected nodes not shown

To better understand proteins and pathways that distinguish MSA from PD/DLB, STRING [59, 60] was used to plot functional and physical interactions between BAR-identified proteins. Each STRING networks' central node (i.e., driver protein) was determined using CentiScaPe 2.2 and highlighted in Fig. 5A, B. Driver proteins (i.e., proteins with the highest “betweenness” score, a node centrality index) for BAR-PSER129 included MAPT (i.e., tau), SNCA, and PRDX1 for PD/DLB, shared, and MSA, respectively. Driver proteins for BAR-MJFR1 included HSP90AB1, GAPDH, and PFN1 for PD/DLB, shared, and MSA, respectively. MCL clustering was conducted to isolate functional networks for each experimental group. Results showed that for PD/DLB BAR-PSER129 identified mostly neuron-specific networks, including SNARE, presynapse, and axon (Fig. 5C), in agreement with our previous observation (Fig. 4). Shared networks for BAR-PSER129, which included SNCA, involved ubiquitin ligase binding, localization, and follin-1-rich granule lumen. A single network of 7 interconnected nodes enriched for peroxidase activity, extracellular exosome, and response to oxidative stress was unique to BAR-PSER129-MSA. For BAR-MJFR1 (Fig. 5D), MSA specific network was also associated with extracellular exosome and peroxidase activity. Physical protein interactions for MSA-proteins were plotted in relation to BAR-target protein (i.e., SNCA). An MSA specific physical subnetwork emerged with peroxiredoxin’s (PRDX1, PRDX2, and PRDX6) as major components. SNCA was segregated from the MSA-specific peroxiredoxin network, with CRYAB, HSPA1B, and HSPA5 connected to SNCA. This BAR-identified subnetwork of MSA was cytosolic (e.g., GAPDH and GFAP), with the regulation of protein stability and response to oxidative stress being the major functional themes.

## Discussion

Here, we interrogated αsyn interactomes within the human brain of two clinically and pathologically distinct primary synucleinopathies. Overall, we found that total αsyn interactions (i.e., BAR-MJFR1) were similar between MSA and PD/DLB brains, with strong enrichment for presynaptic vesicle processes, which is broadly consistent with physiological αsyn interactions, αsyn functionality, and previous proximity proteomics studies [10, 20, 27, 28, 41]. In contrast, αsyn aggregate interactions (i.e., BAR-PSER129) were markedly different between MSA and PD/DLB, with several glial-enhanced proteins (CBR1, CRYAB, and GFAP) (Human Protein Atlas proteinatlas.org [58]) [7] being more abundant in MSA αsyn aggregates (i.e., PD vs. MSA contrast). In addition, five proteins associated with MSA aggregates but also overlapped with the total αsyn pool, including HSPA1B, PRDX1, PRDX2, SEPTIN8, and GSTP1. Collectively identified MSA proteins are functionally interconnected and are involved with anti-oxidative mechanisms and protein stability (Fig. 5E). Multiple oxidative stress mechanisms have been implicated in MSA, including oxidative phosphorylation dysfunction and reductions in anti-oxidative machinery [44]. Reduction in antioxidant glutathione (GSTP1 identified here) may occur in MSA [23, 57], but peroxiredoxins or CBR1 (both proteins protect from oxidative stress) have not been directly implicated in MSA but have been implicated in other neurodegenerative diseases and neurotoxic insults [21, 31]. Together, our findings implicate unique anti-oxidative mechanisms for MSA pathology. We cannot infer precisely how these proteins fit the MSA pathological process, whether protective or toxic. Interestingly, reductions in antioxidant COQ10 have been implicated in MSA pathogenesis [3, 40, 54], albeit inconsistently [49], and clinical efficacy trials for ubiquinol are currently underway [38]. Notably, PFN1 was a central protein for MSA total synuclein; PFN1 is strongly implicated in several neurodegenerative diseases, including amyotrophic lateral sclerosis, frontal temporal dementia, and motor neuron disease [51, 61] [22] [16].

Our data implicates canonical αsyn pathways (i.e., vesicles/SNARE) for PD/DLB and anti-oxidative pathways for MSA but also demonstrates shared interactions between the two distinct synucleinopathies. All experimental conditions shared 26 proteins involved in cellular functions such as intracellular iron sequestering, protein refolding, and nucleoside diphosphate phosphorylation, representing the core interactome of αsyn for both diseases (Additional file 1: Figure S10). At the center of this network was HSPA8, a regulator chaperonin-mediated autophagy inhibitor of αsyn aggregation, which has been implicated in PD, DLB, MSA, and other neurodegenerative diseases [35, 50]. Therefore, our data suggests a protein-refolding response at the center of disparate synucleinopathies. Despite the predominating GCIs in MSA, several neuronal vesicle proteins, including SYNGR3 and SV2A, were identified in the shared pathway (Additional file 1: Figure S10), supporting a neuronal origins hypothesis for MSA. Both proteins could originate in the few PSER129 reactive neuropil in our MSA cases (Fig. 1) or in vesicles released from neurons and endocytosed by proximal oligodendrocytes [33, 39, 48, 70]. Indeed, extracellular exosomes (i.e., L1CAM positive [19, 68]) were consistently enriched pathways in agreement with our previous BAR studies [27], suggesting that αsyn aggregates in synucleinopathy brain partly resemble exosomes and supporting the hypothesis that αsyn/αsyn aggregates can spread via exosomes [11, 15, 34, 63]. It’s unclear whether αsyn “exosomes” have biological importance (e.g., αsyn aggregates are spread by exosomes), but αsyn-containing exosomes have been isolated from peripheral sources [5, 14]. Alternatively, coincidental exosome enrichment may occur because of αsyn aggregates' close association with vesicles/endosomes. Our data suggests one of two scenarios: αsyn aggregates spread to oligodendrocytes via presynaptic neuronal exosomes or, in addition to αsyn, several neuronal proteins are upregulated (SYNGR3 and SV2A) in diseased oligodendrocytes, but this is unlikely because differentiating oligodendrocytes do not express these genes [7]. Overall, our findings support a hypothesis for the potential late-stage contribution of oligodendrocytes to the disease process of MSA (i.e., secondary to neuronal-derived αsyn pathology) [32, 48].

We identified CRYAB associated with GCI’s. CRYAB is upregulated in the MSA brain and directly associated with GCIs [45]. Recent studies have identified GCI-like structures in oligodendrocytes of PD patients carrying αsyn gene mutations or presenting atypical clinical features [69]. CRYAB is encoded by a subpopulation of oligodendrocytes that is depleted in sporadic PD [36]. Functionally, CRYAB binds to the ends of αsyn fibrils, inhibiting aggregation and potentially playing a protective role. The strong association of CRYAB with GCI-related diseases, such as MSA, suggests that this “capping” mechanism plays a prominent role and may contribute to the particularly aggressive clinical progression observed in MSA. Additionally, MSA-derived seeds extracted from the human brain exhibit significantly higher templated-mediated seeding efficiency, which may explain the pronounced CRYAB upregulation and its sequestration to αsyn inclusions. These findings support the potential therapeutic utility of small heat shock proteins in managing these neurodegenerative conditions.

The study's limitations include that most of the MSA cases here are MSA-Parkinsonian type (MSA-P), which is the dominant subtype of MSA in the western hemisphere [65–67], restricting the comparison of PD/DLB to only one subtype of MSA. Another limitation is that BAR, as a “spatial-omics” [24] technique, has special interpretation considerations, including spatial resolution (~ 50–100 nm radius from BAR target), lack of information about the precise nature of protein–protein interactions (i.e., direct or indirect), and although cell type/cell compartment can be inferred from BAR data, it cannot be determined. Although not explored here, determining how BAR-identified proteins compare to cell-specific (e.g., oligodendrocytes or neuronal proteomes) or organelle-specific proteomes might help better understand interactions within cellular contexts. Additionally, although our analysis focused on MSA, the networks/proteins specific to PD/DLB identified here are likely valuable for follow-up analyses aimed at deciphering PD/DLB mechanisms.

When interpreting BAR-PSER129 data, an important consideration should be made, specifically, the association of PSER129 with αsyn aggregates, which is not without caveats. Here, we use PSER129 as an aggregate marker, but physiological non-aggregated PSER129 can occur in the mammalian brain [28, 43, 46], and thus, PSER129 is not an implicit marker of aggregates. However, we recently discovered that physiological non-aggregated PSER129 is rapidly dephosphorylated during post-mortem intervals [8]. Correspondingly, post-mortem specimens used here likely contain mostly aggregated-PSER129. This conclusion is consistent with PSER129 staining here, which lacked distribution and GM abundance characteristic of the mammalian brain [28]. Nevertheless, it remains possible that some BAR-determined interactions are from physiological PSER129, and not αsyn aggregates.

Proximity proteomics are powerful techniques used to study αsyn aggregate and non-aggregate interactomes [27], but this is the first application of BAR comparing synucleinopathies and there currently aren’t standardized data analysis pipeline specifically for BAR. As we found before [27], background captures (BAR-NEG) were a critical component of the study design that allowed the identification of BAR-antibody-specific proteins. Direct comparisons between diseases (e.g., MSA vs PD/DLB) could be made for each BAR capture, but background proteins must be removed manually, as background (BAR-NEG) information is lost in this approach. To increase protein identification and robustness of our analysis we conducted two analyses based on two quantitative values LFQ scores (Maxquant) and TNS (Scaffold). We found a correlation between methods (Additional file 1: Figure S5, S6), but LFQ was superior in that the target protein (αsyn) was consistently the most enriched protein, in contrast to TNS, where αsyn was only most abundant in the MSA-PSER129 capture (Additional file 1: Figure S3). Additional analysis might be fruitful in further elucidating the significance of BAR-identified proteins, including comparison with complementary datasets and incorporating analysis with different experimental assumptions (Additional file 1: Figure S11-14, Additional file 11).

## Conclusions

In conclusion, the results suggest that MSA involves PRDX's anti-oxidative processes in glial cells, while presynaptic vesicle/SNARE processes dominate PD/DLB. Our findings revealed a significant overlap between MSA and PD/DLB, suggesting shared pathogenic origins for these synucleinopathies. Intriguingly, we identified several neuronal proteins, besides SNCA and strong exosome enrichment, associated with GCIs, together supporting the neuron-to-glia spread hypothesis of MSA.

## Supplementary Information

Additional file 1.

Additional file 2.

Additional file 3.

Additional file 4.

Additional file 5.

Additional file 6.

Additional file 7.

Additional file 8.

Additional file 9.

Additional file 10.

Additional file 11.

Additional file 12.

Additional file 13.

## Data Availability

The mass spectrometry proteomics data have been deposited to the ProteomeXchange Consortium via the PRIDE partner repository with the dataset identifier PXD05530.

## Abbreviations

NI: Neuronal inclusions
GI: Glial inclusions
LFQ: Label free quantification
TNS: Total normalized spectra
BAR: Biotinylation by antibody recognition
NEG: Primary antibody omission
MJFR1: Antibody raised against αsyn c-terminus
PSER129: αSyn phosphorylated at serine 129
PCA: Principal component analysis
PD: Parkinson’s disease
DLB: Dementia with lewy bodies
MSA: Multiple system atropy
Αsyn: Alpha-synuclein
GCI: Glia cytoplasmic inclusion
PDD: Parkinson’s disease dementia
DM: Dilution media
HIAR: Heat induced antigen retrieval
BSA: Bovine serum albumin
ABC: Avidin biotin complex
DAB: 3,3'Diaminobenzidine
DA: Differential Abundance
FDR: False discovery rate
GO: Gene ontology
PVDF: Polyvinylidene fluoride
ECL: Enhanced chemiluminescent reagent
WM: White matter
GM: Gray matter
IHC: Immunohistochemistry
WB: Western blot
