# Supplementary material for: Proximity proteomics reveals unique and shared pathological features between multiple system atrophy and Parkinson’s disease
Source: Acta Neuropathol Commun. 2025 Mar 23;13:65. doi: 10.1186/s40478-025-01958-5 (PMC11931798; doi:10.1186/s40478-025-01958-5)
Supplement: Supplementary file 1 — Additional file 1. [file 40478_2025_1958_MOESM1_ESM.pdf]

Supplemental Information for:

Proximity proteomics reveals unique and shared pathological features between multiple system atrophy and Parkinson’s disease

[1]Choi S.G., [1]Tittle T., [4]Barot, R, [3]Betts, D., [1]Gallagher J., [2]Kordower J.H., [2]Chu Y., [1]Killinger B.A.

[1]Department of Neurological Sciences, Rush University Medical Center, Chicago, IL, USA.

[2]ASU-Banner Neurodegenerative Disease Research Center and School of Life Sciences, Arizona State University, Tempe, AZ, USA.

[3]University of Michigan, Ann Arbor, MI, USA.

[4]University of Illinois at Chicago. Chicago IL, USA.

Table of Contents:

Figure S1- Thresholding midbrain and forebrain images
Figure S2- Tissue weights used for experiments.
Figure S3- Volcano plots for TNS method.
Figure S4- Top ten Driver pathways.
Figure S5- Comparison of LFQ and TNS methods for BAR-PSER129.
Figure S6- Comparison of LFQ and TNS methods for BAR-MJFR1.
Figure S7- Heatmap for TNS method.
Figure S8- Correlation heatmap for TNS method.
Figure S9- PCA plot for TNS method.
Figure S10-STRING network for 26 proteins common to all captures and disease states.
Figure S11. Proteins exclusive to BAR-capture conditions.
Figure S12. STRING functional interactions of capture exclusive identified proteins.
Figure S13.STRING functional interactions of capture exclusive identified proteins.
Figure S14. Cell-type of BAR-identified proteins in MSA brain.

Supplemental data sets:

Additional file 2. DEP results for LFQ method.
Additional file 3. DEP results for TNS method.
Additional file 4. List of significant proteins identified in DEP analyses. Non-ranked.
Additional file 5. = MSA.MJFR1 GODriver enrichment file.
Additional file 6. = MSA PSER129 GODriver enrichment file.
Additional file 7. = PDDLB GODriver enrichment file.
Additional file 8. = PDDLB PSER129 GODriver enrichment file.
Additional file 9 = Input.Scaffold.Mascot.TS.xls
Additional file 10 = Input.MAXquant.LFQ.xls”
Additional file 11. “Exclusive analysis” protein lists.
Additional file 12. Maxquant parameters file.
Additional file 13. Scaffold/mascot parameters file.

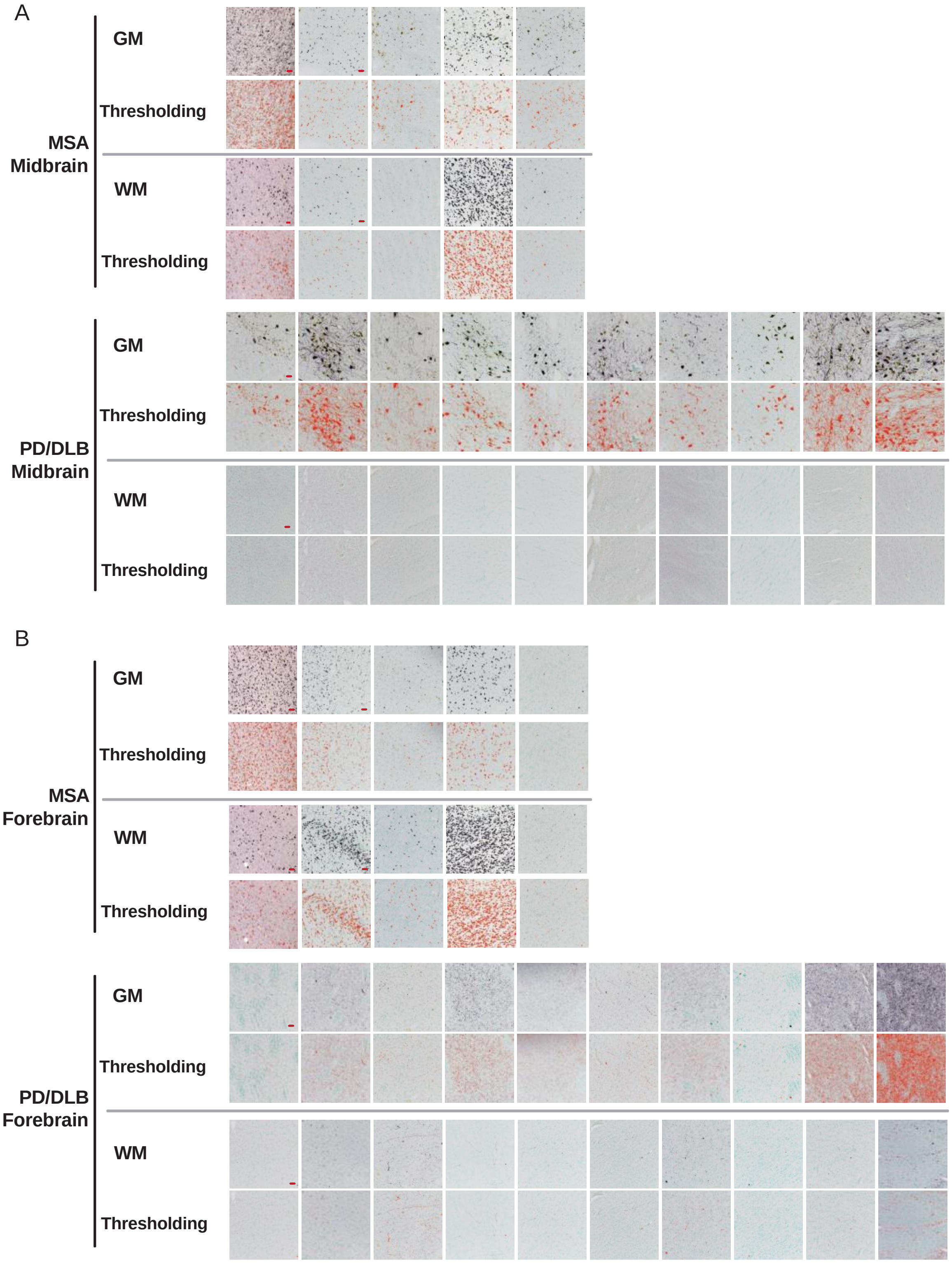

Figure S1. Thresholding midbrain and forebrain images. To quantify the PSER129 signals in each brain region, images were acquired from (A) the midbrain and (B) the forebrain of MSA and PD/DLB brain. Nikon elements thresholding algorithm was applied to batches of images using the same settings for each batch (Threshold result appears red). Grey matter (GM) images were taken at the SNpc in the midbrain, while white matter (WM) images were captured in the white matter tract near the GM. For the forebrain, GM images were taken from the putamen, and WM images were taken at the WM tract near the putamen. Scale bars = 50 μm.

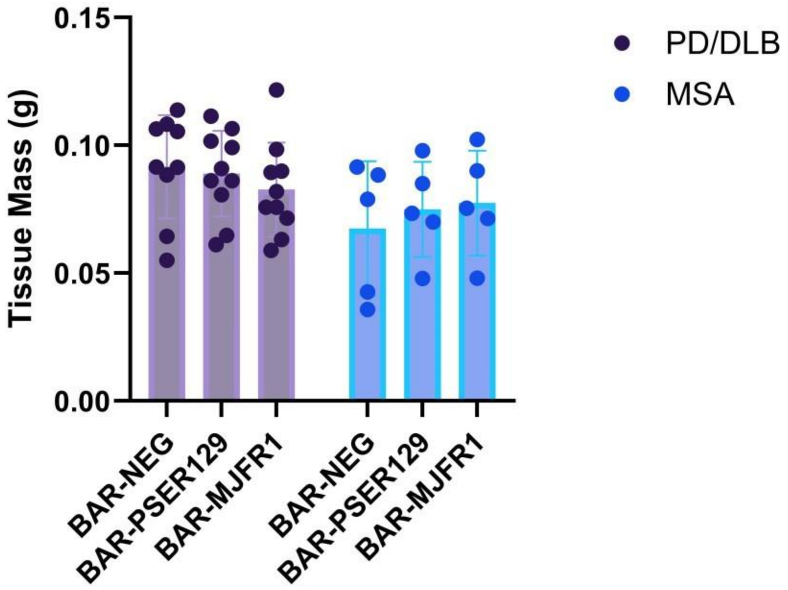

Figure S2. Tissue weights used for BAR experiments. A single forebrain and midbrain section were pooled for each BAR capture. Before BAR, wet tissues were weighed for each capture. The graph shows the tissue weights for all BAR samples prepared. PD/DLB, n=10 and MSA, n= 5.

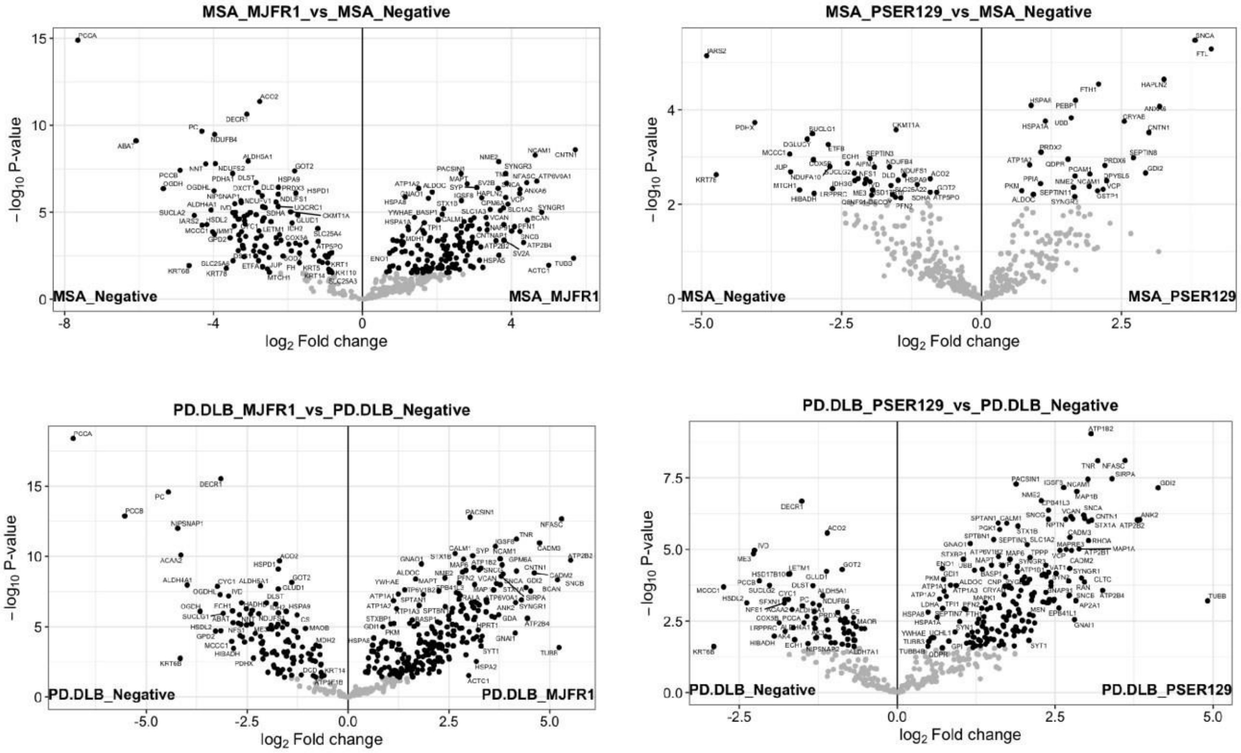

Figure S3. Volcano plots for TNS method. Plots display the proteins quantified for each condition using the TNS method. For BAR-PSER129 in MSA, SNCA was the most enriched protein, while other captures included SNCA above the background but did not rank it as the top hit.

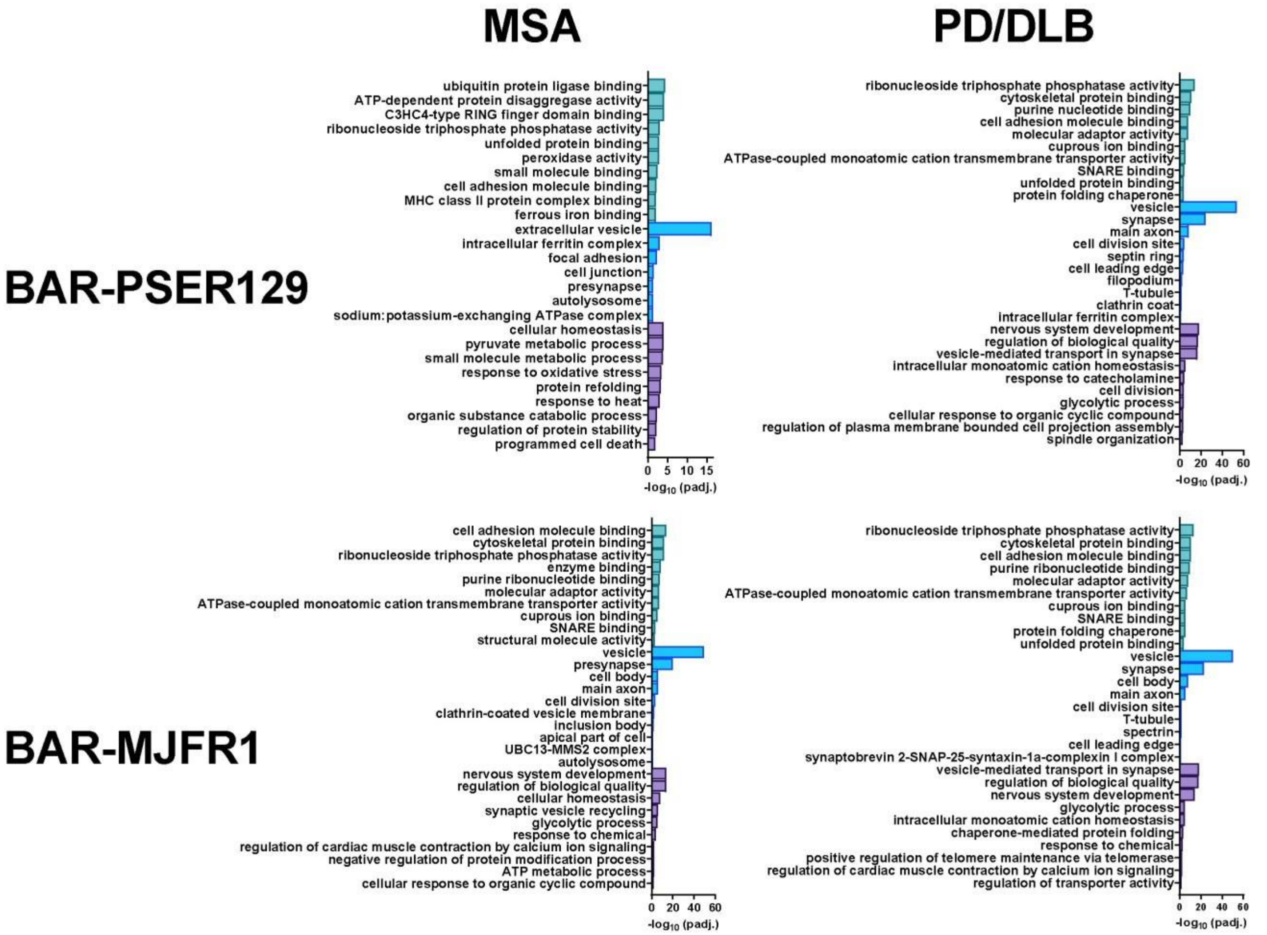

Figure S4. Top-10 enriched GO pathways. gProfiler enrichment was conducted on BAR-identified proteins. Graphs show the top 10 (ranked by padj.) driver GO pathways for each condition. All driver GO pathways are found in the enrichment map, Figure 4.

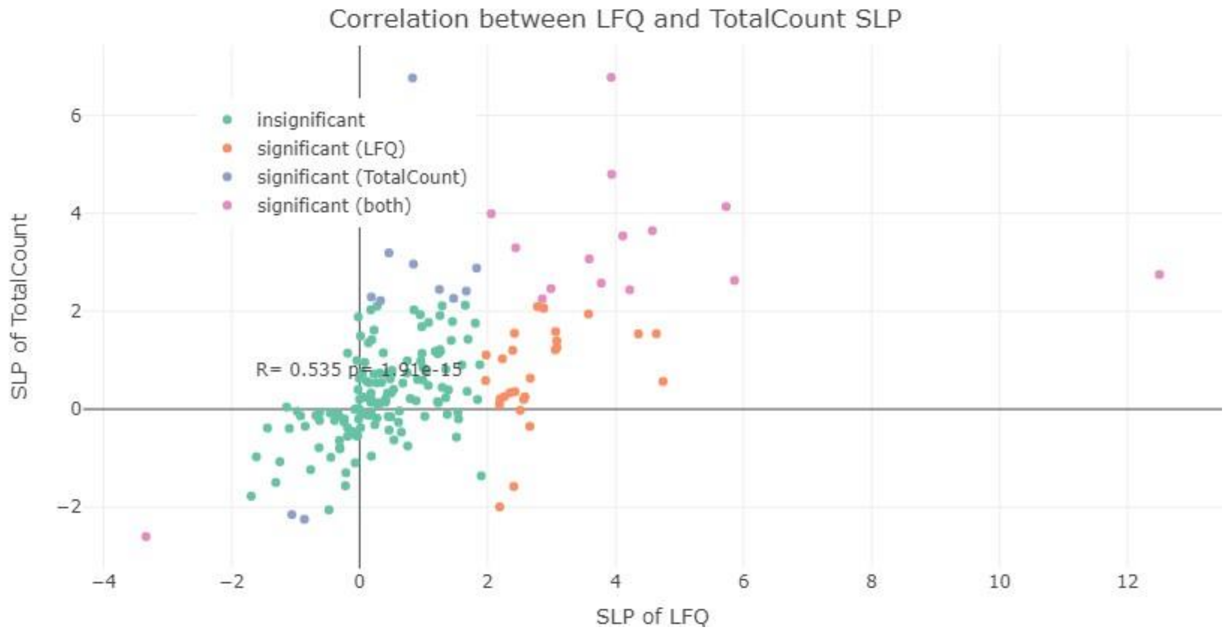

Figure S5. **Comparison of LFQ and TNS ("total count") methods for BAR-PSER129**. Sine log-p-value (SLP) was calculated for differential abundance between LFQ and TNS methods. This method takes p-value and log fold-change into account. Only proteins captured in both analyses are included in the comparison. Pearson R was calculated and displayed on the graph.

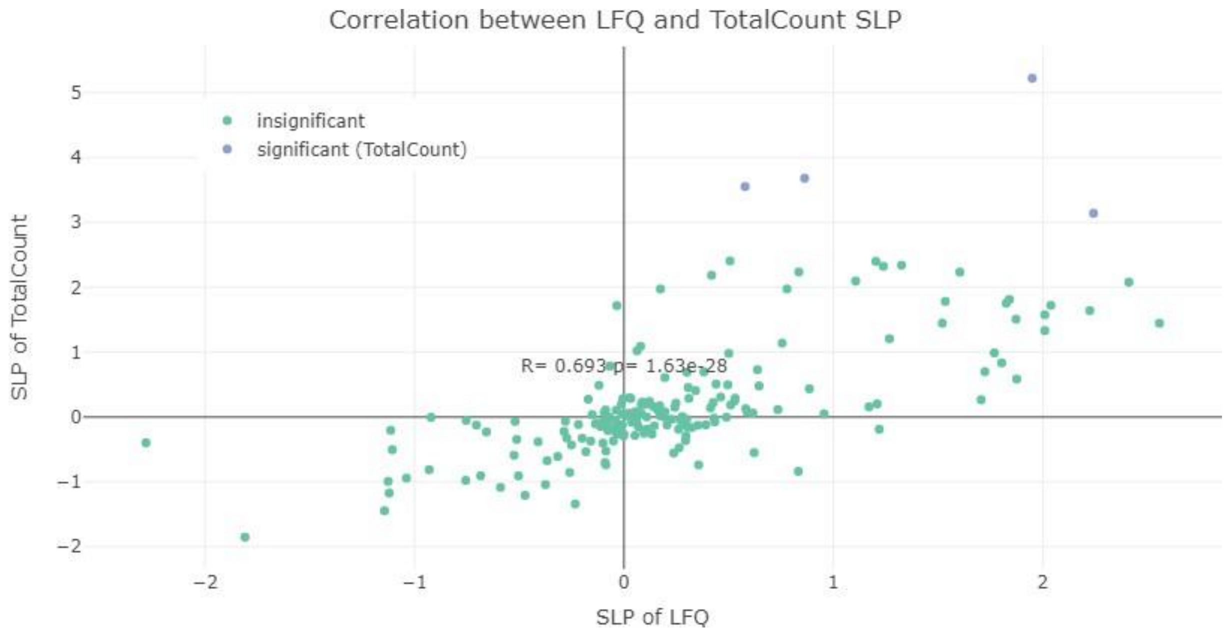

Figure S6. Comparison of LFQ and TNS ("total count") methods for BAR-MJFR1. Sine log-p-value (SLP) was calculated for differential abundance between LFQ and TNS methods. This method takes p-value and log fold-change into account. Only proteins captured in both analyses are included in the comparison. Pearson R was calculated and displayed on the graph.

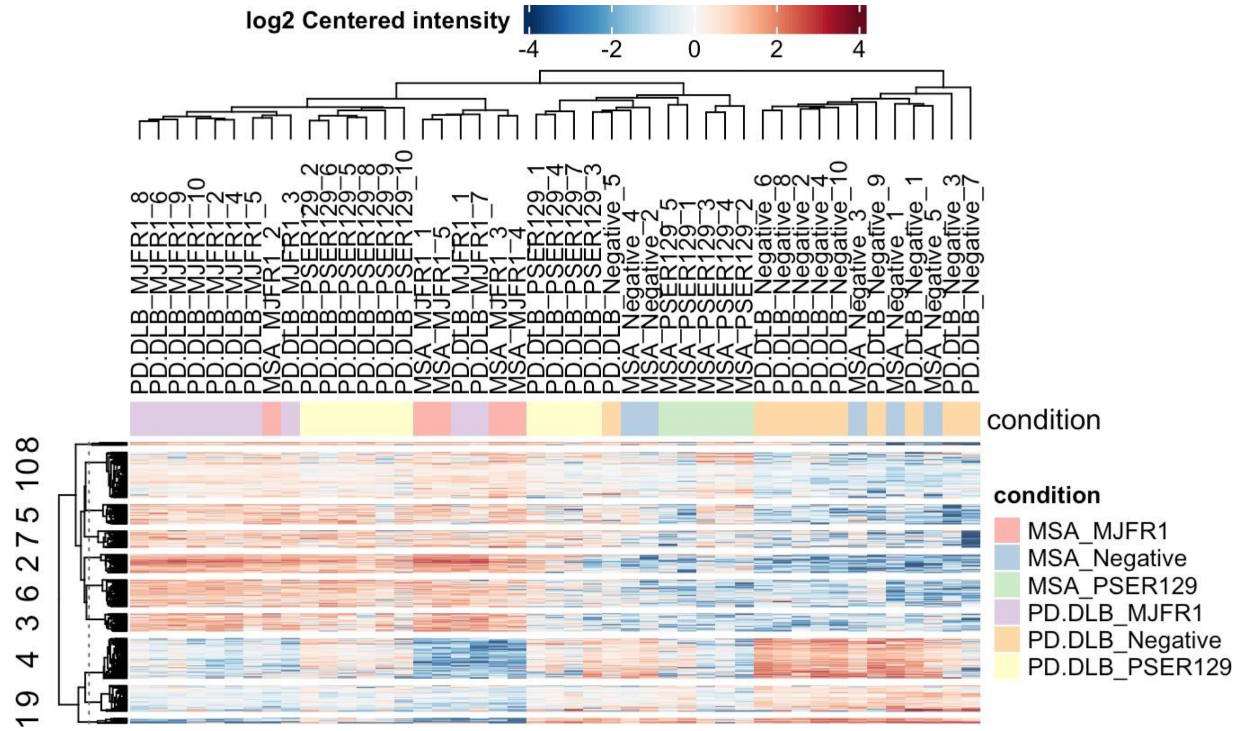

Figure S7. Heatmap for TNS method. Hierarchical clustering of protein abundance among all tested samples. The cases formed two main clusters: one cluster included BAR-NEG for both MSA and PD/DLB, while the other main cluster showed strong “on-target” signals for BAR-MJFR1 in MSA and PD/DLB, as well as for BAR-PSER129 in PD/DLB. Although BAR-PSER129 in MSA was included in this cluster, it exhibited weaker “on-target” signals.

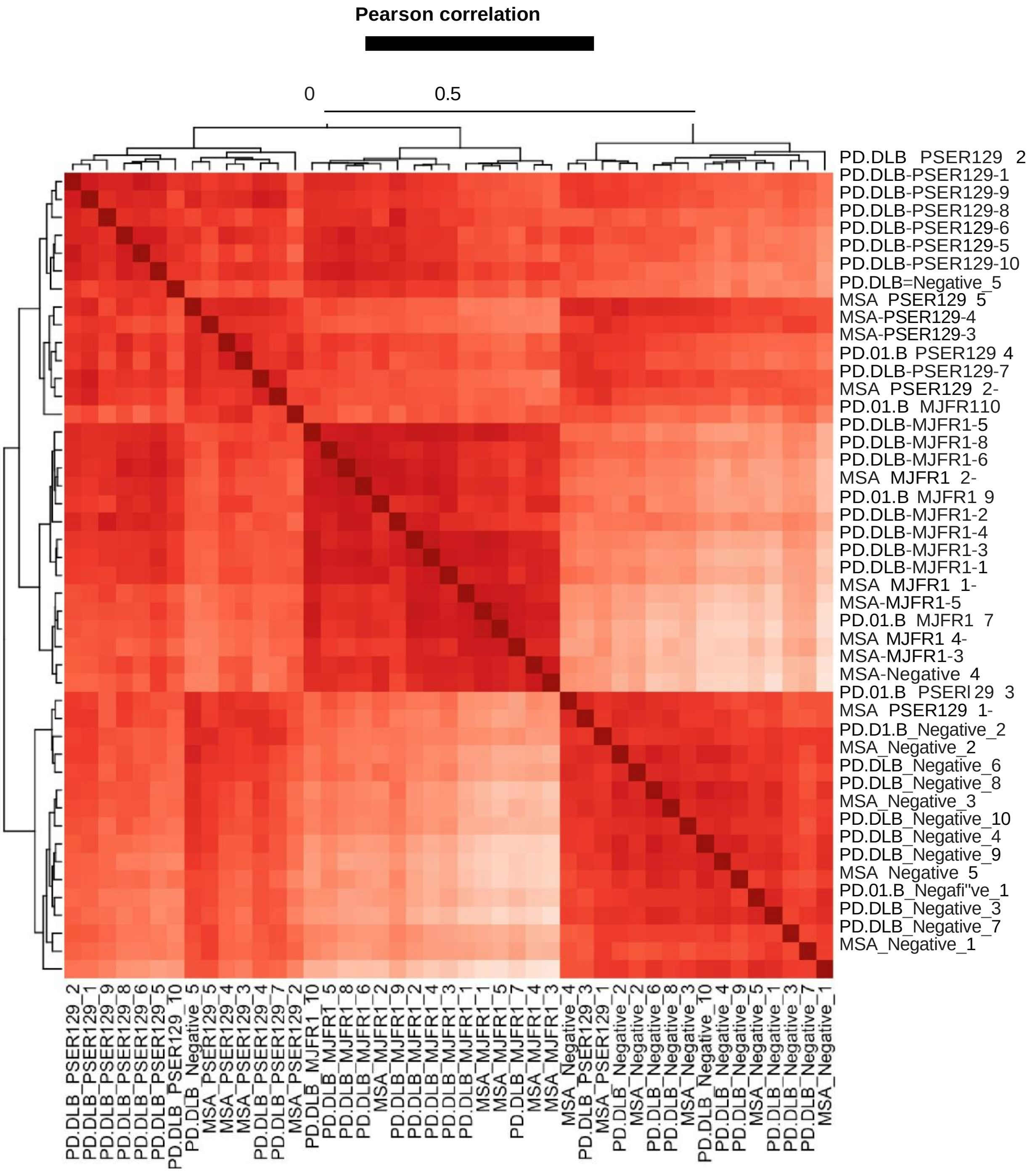

Figure S8. Correlation heatmap for TNS method. Hierarchical clustering of Pearson correlations among all tested samples. BAR-MJFR1 showed a close correlation between MSA and PD/DLB, as did BAR-NEG between the diseases.

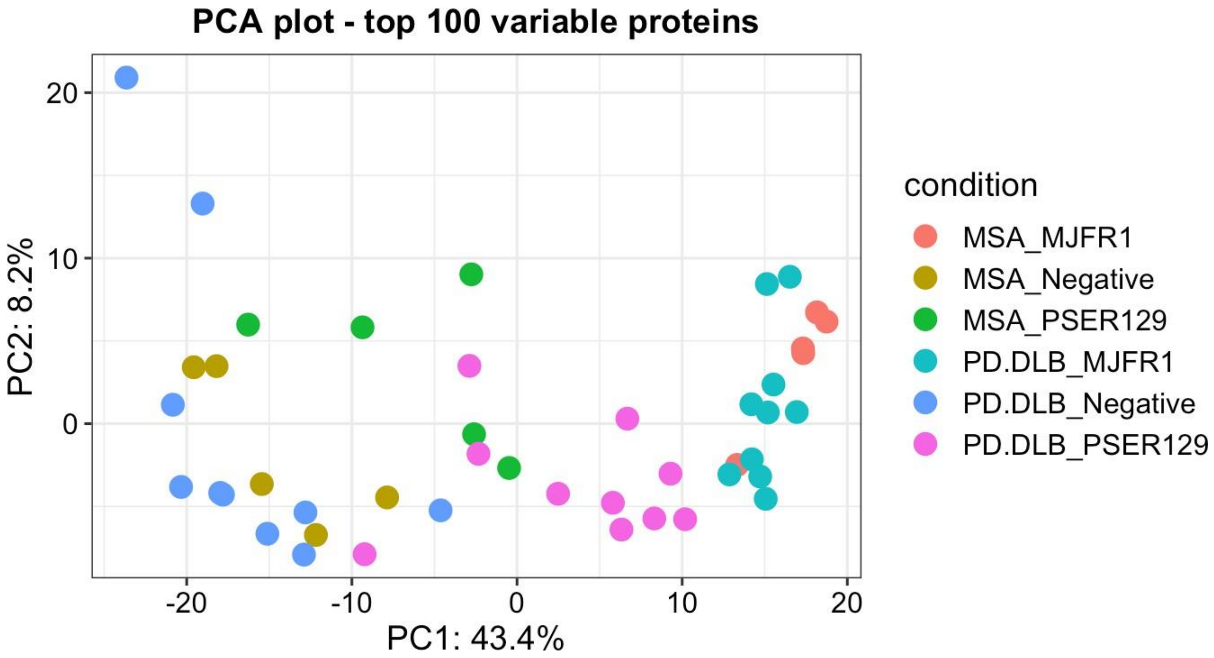

Figure S9. PCA plot for TNS method. PCA plot for the TNS method, which displays the top 100 BAR-captured proteins, reveals that BAR-MJFR1 in MSA and PD/DLB exhibit close groupings, overlapping in both PC1 and PC2. BAR-PSER129 for MSA is positioned near the BAR-NEG captures, while the captures in PD/DLB are close to the BAR-MJFR1 captures.

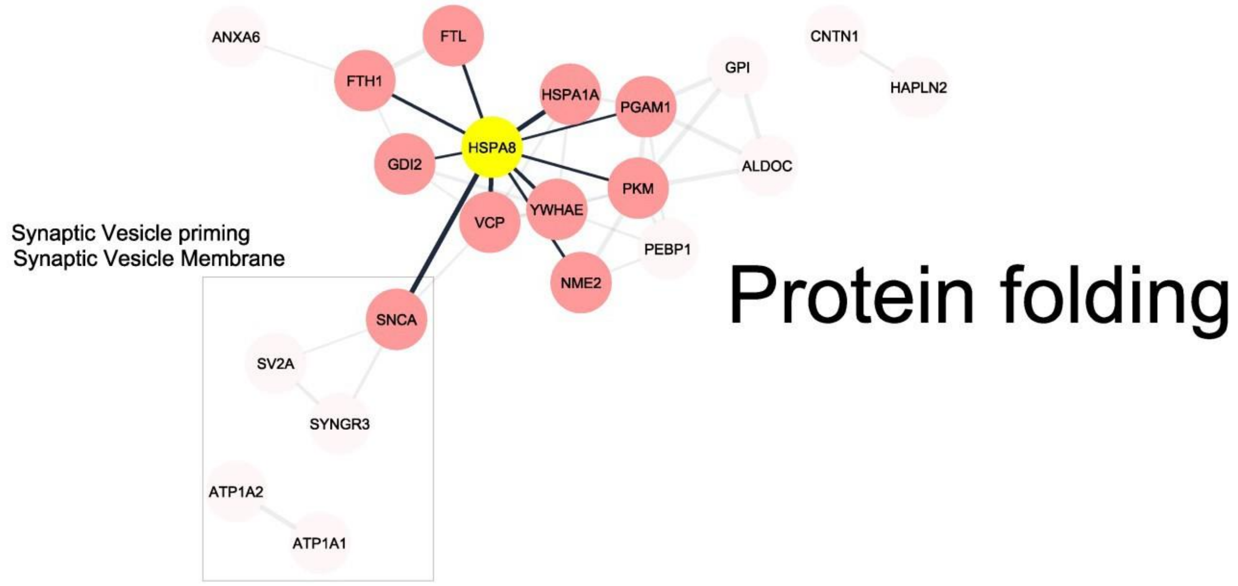

Figure S10. STRING network for 26 proteins common to all captures and disease states. 26 proteins were identified and enriched for all captures and disease states. STRING network of these proteins reveals a network with HSPA8 as the central node and pathways involving protein folding as the major enrichments. Interestingly, several presynaptic proteins (SV2A and SYNGR3) are included in this network despite the lack of neuronal pathology observed in MSA.

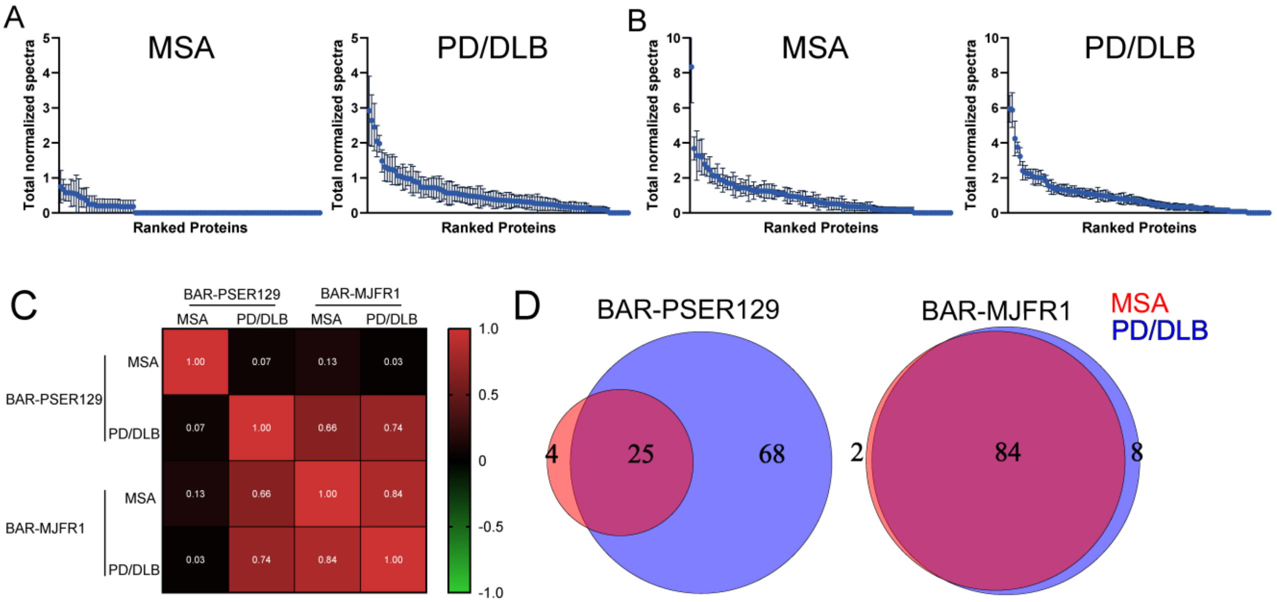

Figure S11. Proteins exclusively identified in BAR-capture conditions. Scaffold/mascot-identified proteins (TNS method, Additional File 9) never identified in BAR-NEG condition (i.e., exclusive to BAR capture) were determined and analyzed. Keratins were removed, and those proteins were separated according to experimental conditions they were detected in (i.e., BAR-PSER129 or BAR-MJFR1; PD/DLB or MSA). Protein lists generated using these parameters are found in Additional file 11. Proteins ranked by the sum TNS for (A) BAR-PSER129 and (B) BAR-MJFR1 across disease conditions. (C) Correlation matrix of Pearson correlation coefficient values when comparing the ranked exclusive proteins between all conditions. (D) Venn diagrams showing overlapping and proteins unique to each experimental condition. A-C generated using GraphPad prism 10.4.1. D generated using Venn Diagram Generator (barc.wi.mit.edu/tools/venn/).

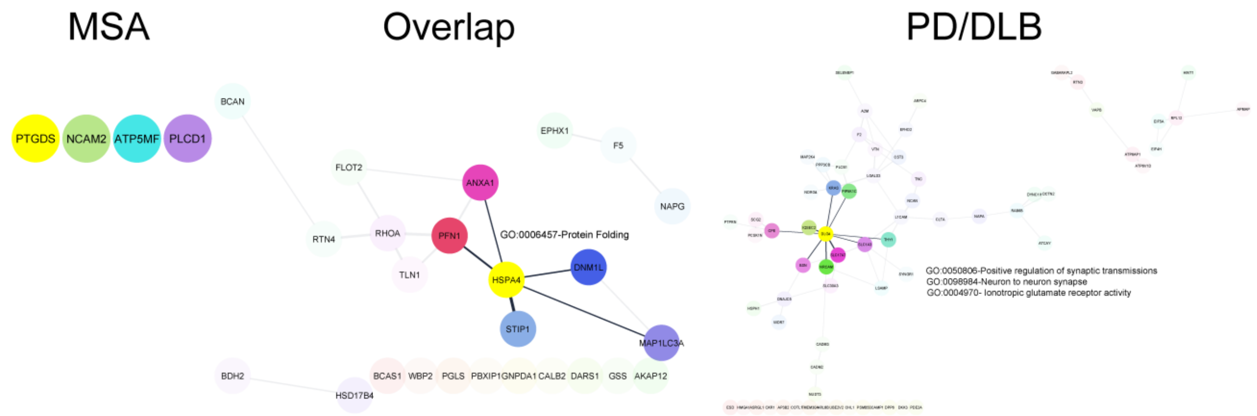

Figure S12. STRING functional interactions of capture exclusive identified proteins. BAR-PSER129 identified proteins from Fig. S11 were mapped using STRING as described in the methods. Singleton nodes are shown. Central node and immediate interactors are highlighted where applicable. Networks annotated with top GO Ontology terms or top significant enrichment terms when GO Ontology enrichment wasn't found.

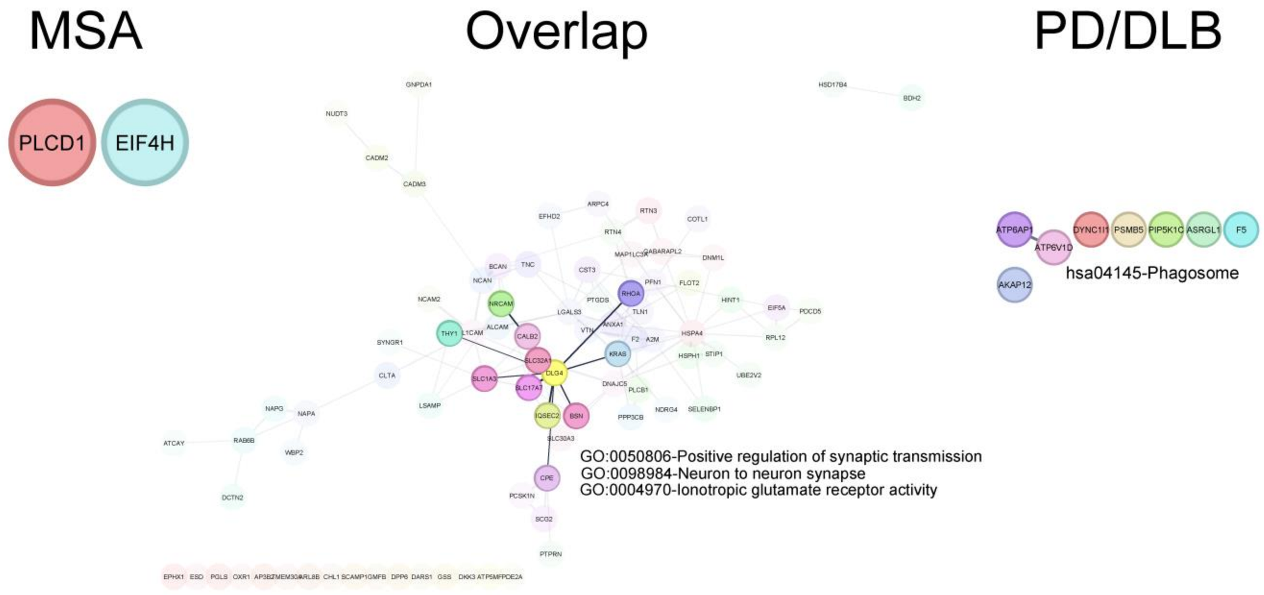

Figure S13.STRING functional interactions of capture exclusive identified proteins. BAR-MJFR1 identified proteins from Fig S11 were mapped using STRING as described in the methods. Singleton nodes are shown. Central node and immediate interactors are highlighted where applicable. Networks annotated with top GO Ontology terms or top significant enrichment terms when GO Ontology enrichment wasn't found.

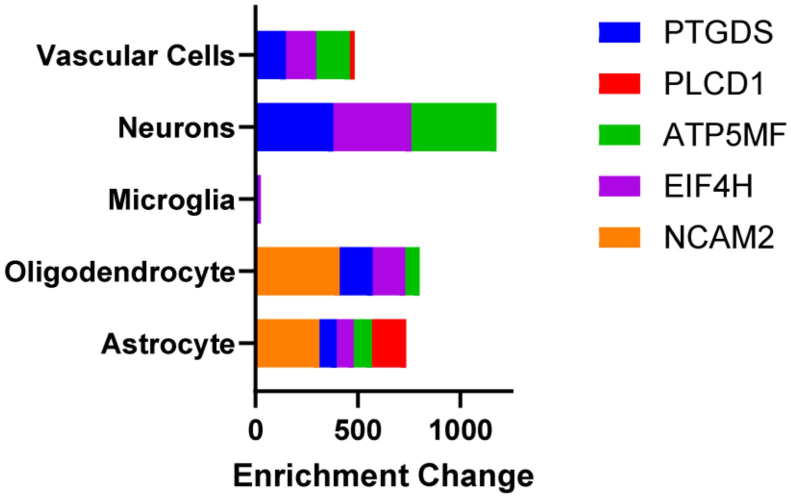

Figure S14. Cell-type of BAR-identified proteins in MSA brain. Spatial transcriptomic data from the human protein atlas (www.proteinatlas.org) was used to estimate the cellular origins of each BAR-identified protein from Figure S11. Stereo-seq enrichment scores for each protein were plotted. Values are plotted as part of a whole. Thus, the strength of association for each cell type can be estimated. PLCD1 was the only protein lacking association with oligodendroglia. All proteins can be found in astrocytes.
